# Short term ex vivo graft conditioning with venetoclax, fluticasone propionate, and IL-2 reduces graft versus host disease in mouse allogeneic hematopoietic cell transplantation

**DOI:** 10.3389/fimmu.2026.1746010

**Published:** 2026-09-24

**Authors:** L. Angel Ayala, Alizeh Ahmedani, Cuiwen Zhou, Gabriella L. Hisoire, Alexii Villamar, Michelle Baez, Nathalia Onofre, Chanel De Smet, Pauline U. Nguyen, Vanessa M. Scarfone, Erika S. Varady, Xiyu Chen, Ankita Daud, Matthew A. Inlay

**Affiliations:** 1 Department of Molecular Biology and Biochemistry, University of California, Irvine, Irvine, CA, United States; 2 Sue and Bill Gross Stem Cell Research Center, University of California, Irvine, Irvine, CA, United States

**Keywords:** allogeneic hematopoietic cell transplantation, glucocorticoid, graft-versus-host disease, regulatory T cell, venetoclax

## Abstract

Hematopoietic cell transplantation is a curative therapy for certain hematologic malignancies but is limited in use due to post-transplant complications such as graft-versus-host disease (GVHD), wherein donor cells mount an immune response against host tissues. GVHD is a major cause of post-transplant mortality, inspiring the development of prophylactic strategies to reduce GVHD severity. However, these involve administration of cytotoxic or immunosuppressive compounds into patients, increasing adverse side effects. To address this issue, we have developed a strategy to reduce GVHD through ex vivo graft conditioning. Previously we demonstrated that pretreatment of mouse grafts with a single compound, fluticasone propionate, dramatically reduced GVHD symptoms after allogeneic transplantation. Here, we condition grafts in a combination of fluticasone propionate, venetoclax, and IL-2 for 6 hours ex vivo prior to transplantation. We demonstrate that the preconditioning process increases the frequency of tolerogenic regulatory T cells pre- and post-transplant. Recipient mice receiving conditioned cells display reduced GVHD severity and increased overall survival in both fully mismatched and haploidentical models. This study highlights an alternative strategy to GVHD prophylaxis which reduces reliance on the administration of toxic compounds.

## 1 Introduction

Hematopoietic cell transplantation (HCT) is a potentially curative therapy to address a variety of blood disorders, including leukemias, lymphomas, aplastic anemia and immune-deficiency disorders (1). HCT from a non-self donor is referred to as allogeneic (allo)-HCT and accounts for 40-50% of total HCT transplants performed over the last several years (2). However, due to the high risk of post-transplant complications, allo-HCT is primarily used to treat leukemia (3). Moreover, allo-HCTs have the added benefit of an anti-tumor response known as graft-versus-tumor (GVT) effect which eliminates cancerous cells (4) and is primarily carried out by CD8+ cytotoxic T cells (5, 6).

A major complication following allo-HCT is acute graft-versus-host disease (aGVHD) wherein donor immune cells recognize the patient’s tissues as foreign and mount an immune response causing morbidity and mortality. Following allo-HCT, 30-70% of recipients develop acute GVHD due primarily to a partial mismatch of the human leukocyte antigen (HLA) genes between donor and patient (7). Immunosuppressive glucocorticoid (GC) administration has long been the standard of care after the onset of GVHD symptoms. However, long term administration of GCs has been associated with increased rates of opportunistic infection, diabetes and myopathy contributing to mortality and morbidity (8). In addition, prophylactic treatment of allo-HCT recipients with GCs prior to the onset of GVHD symptoms was not beneficial due in part to the increase in complications resulting from enhanced immunosuppression in already-immunocompromised patients (9, 10).

Regulatory T cells (Tregs, CD4+, FoxP3+) are a rare subclass of CD4+ T cells with immunosuppressive properties and play an important role in maintaining tolerance, preventing autoimmunity, and blocking alloreactivity upon transplantation. Mouse Tregs transplanted in a 1:1 ratio with conventional T cells (Tcons, CD4+, FoxP3-) are able to reduce GVHD while maintaining GVT in a fully mismatched mouse model of GVHD (11). Although Tregs have an important in role in suppressing GVHD, transplanting Tregs and Tcons at a 1:1 ratio clinically would require the sorting of cells via fluorescent activated cell sorter (FACS), or ex vivo expansion, and in both cases would require use of a GMP facility, a costly and time-consuming step, and thus is less practical for widespread adoption. To address this, our lab has developed a method to reduce aGVHD severity in a fully mismatched GVHD mouse model through a strategy we term *ex vivo* graft conditioning (EVGC).

This process involves conditioning donor cells with a GC, fluticasone propionate (FLU) for 16 hours prior to transplantation. This results in a reduction in Tcon viability which leads to an approximately 6-fold increase in the frequency of Tregs (3% to 18%), a frequency which persists *in vivo* for at least 10 days post-transplantation. Importantly, 50% of mice given FLU grafts survived to 120 days, whereas all mice given vehicle grafts died within 28 days (12). It is important to note that FLU was not given directly to the recipient, only to the donor cells *ex vivo* prior to transplantation, thus avoiding potential side effects of GC administration to the recipients. While 50% survival is a dramatic improvement from 0% for a fully mismatched transplantation, it leaves substantial room for improvement. In this study, we explore the use of additional compounds to further reduce Tcon viability and increase the Treg: Tcon ratio to 1:1.

Tregs express IL-2 receptor alpha (CD25) at steady state, and their survival depends critically on IL-2 signaling. Tcons and CD8+ T cells only upregulate CD25 upon activation. Thus, in non-activating conditions, IL-2 will only stimulate Tregs to survive and proliferate. Venetoclax (VEN) is a BH3 mimetic small molecule inhibitor of the anti-apoptotic protein Bcl-2, and can synergize with other compounds to induce apoptosis in tumor cells. Here, we combine treatment of splenic T cells with FLU, VEN, and IL-2 to eliminate most Tcons and CD8+ T cells, while preserving Tregs, to create an ex vivo graft conditioning protocol. As such, we were able to achieve a 1:1 ratio of Tregs to Tcons in as little as 6 hours, and show improvements to GVHD clinical scores and survival beyond what we could achieve with FLU treatment alone.

## 2 Materials and methods

### 2.1 Mouse lines

B6.Cg-*Foxp3^tm2Tch^*/J (“B6-FoxP3^EGFP^”, or “FoxP3-GFP”, H-2^b^, The Jackson Laboratory, #006772). BALB/cJ (“BALB/c”, H-2^d^ background, The Jackson Laboratory, #000651). C3H/HeJ (“C3H”, H-2^k^, The Jackson Laboratory, #000659). CFP+ (H-2^b^, a generous gift from Irving Weissman). All mice were maintained at University of California Irvine: Gross Hall and Medical Sciences A vivaria. Mice were fed standard chow unless otherwise stated. All procedures performed on mice were approved by the International Animal Care and Use Committee (IACUC) and the University Laboratory Animal Resources (ULAR) team at the University of California, Irvine.

### 2.2 *Ex vivo* graft conditioning

We previously published detailed protocols for harvesting tissues, graft conditioning and flow cytometric analysis (13, 14). Fresh splenocytes were harvested from FoxP3-GFP (H-2^b^) mice by dissecting and grinding whole spleens between frosted glass microscope slides into X-VIVO media (X-VIVO 15, Lonza/Fisher Scientific, #BW04-418Q). Homogenized splenocytes were centrifuged and resuspended in 1x ACK lysis buffer (Cytek Biosciences, #TNB-4300-L100) and incubated at room temperature for five minutes to lyse red blood cells. Following centrifugation and resuspension in X-VIVO media, cells were filtered through 64µm mesh (Genesee Scientific, #57-104). 1 x 10^6^ donor H-2^b^ splenocytes were cultured in 24-well plates in 500 uL of X-VIVO media per well, and treated (i.e. “conditioned”) in either vehicle (0.5% DMSO), 3nM fluticasone propionate (Sigma Aldrich, #F9428), 1, 10, or 100nM Venetoclax (Fisher Scientific, #50-136-6419), and/or 20 ng/mL recombinant human IL-2 (Biolegend, #589104) and incubated at 37 °C in 5% CO_2_. After cell culture, wells were washed and stained for flow cytometric analysis or collected for transplantation. Conditioned cells for transplantation were only counted prior to cell culture. Cell numbers were not adjusted post-culture.

### 2.3 Flow cytometry

For external surface staining, cells were stained with antibodies in 25–50 uL of FACS buffer (2% fetal bovine serum in PBS) for 15 min on ice, then washed in FACS buffer and then incubated for 5 min at room temperature with Annexin V (Biolegend #640912) in Annexin binding buffer (Biolegend, #422201), followed by addition of propidium iodide and 10, 000 accudrop beads (for cell counting, BD Biosciences, #345249) in Annexin binding buffer. Cells were analyzed on the BD Fortessa X20. Viability was measured by cells negative for both propidium iodide (stains dead cells) and Annexin V (stains apoptotic cells). Treg percentage was determined by the reporting of GFP from FoxP3-GFP cells in live, CD3+, CD4+ cells. For Intracellular staining, the Foxp3/Transcription Factor Staining Buffer Kit (Cytek Biosciences, #TNB-0607-KIT) was used following the manufacturer’s instructions. Briefly, cells were first stained with Zombie Red fixable viability dye (1:200, Biolegend, #423109) in PBS for 5 min at room temperature, followed by washing in FACS buffer, then 1x Fix/Perm buffer was added for 20 min at 4 °C, transferred to FACS tubes, and washed in 1x Perm buffer. After fixation, cells were centrifuged in FACS tubes at 800 x g. All antibodies were diluted 1:200 in 1x Perm buffer and stained overnight at 4 degrees. The full antibody panel can be found in **Supplementary Table 1**. To estimate absolute numbers of cells, 10, 000 accudrop beads were added to each sample prior to flow cytometry analysis. To calculate absolute number: First divide 10, 000 (beads) by the number of beads collected and multiply by number of events collected for viable T cell subsets.

(e.g. 10, 000 beads/1000 beads acquired X 5, 000 viable T cells acquired = 50, 000 T cells in sample).

### 2.4 Transplantation

5 x 10^6^ cultured splenocytes (counted pre-culture) and 1 x 10^6^ uncultured BM cells from a FoxP3-GFP mouse were resuspended in 100 µL of FACS buffer and injected retro-orbitally into sex/age matched, lethally irradiated (800 cGy) BALB/c (H-2^d^) recipients. Mice were anesthetized with isoflurane. To protect from infection, mice were fed antibiotic chow (Uniprim, Envigo Bioproducts, #NC0900829) for 3 weeks post-transplantation. Each transplantation and subsequent grading period was performed as a blinded study, the information about culture condition was unknown to the researcher until after completion of analysis.

### 2.5 Post-transplant intracellular cytokine staining and T cell stimulation

To assess T cell subsets following transplantation, spleens were harvested from recipients 10 days post-transplantation. Splenocytes from each mouse were then divided into three aliquots. One aliquot was used to stain for extracellular markers, another was plated onto a 24-well plate and underwent a T cell stimulation, and the last aliquot was plated without T cell stimulation. Cultured cells were either unstimulated in X-VIVO media or stimulated by adding the Cell Stimulation Cocktail in a 1:500 dilution (500X, Tonbo Biosciences, #TNB-4975). The cocktail includes PMA (Phorbol 12-Myristate 13-Acetate, 81 nM), Ionomycin (1.34 µM), and protein transport inhibitors Brefeldin A (10.6 nM) and Monensin (2 nM) at the indicated final concentrations. Cells were incubated for 4 hours in complete RPMI at 37 °C and 5% CO_2_, then harvested and stained as described in the flow cytometry section.

For the external stain, antibodies were used against the following markers: CD3, CD4, CD8, CD25, and PD1. Donor chimerism was measured by staining with MHC haplotype-specific antibodies for C57BL/6 (H-2^b^) and BALB/c (H-2^d^). For the intracellular stain, stimulated and unstimulated cells were washed with FACS buffer, transferred into FACS tubes and subjected to Foxp3/Transcription Factor Staining Buffer Kit. Cells were analyzed by flow cytometry on the BD Fortessa X20 for CD3, CD4, CD8, CD25, CD45, FoxP3, H-2^b^, H-2^d^, IFN*γ*, IL-4, and IL-17. After gating for live CD4+ T cells (Zombie Red-, CD45+, CD3+, CD4+, CD8-), helper T cell subsets were identified as Th1 (IFN*γ*+), Th2 (IL-4+), Th17 (IL-17+), and Treg (FoxP3+). For identifying Tregs, the unstimulated cells were used as FoxP3 downregulates in stimulated T cells.

### 2.6 GVHD grading

Clinical scores were recorded daily for the first 12 days post-transplant according to a previously established protocol (Naserian et al., 2018). Mice were observed for signs of GVHD including dull fur, hunched back, skin lesions, diarrhea, and 10% loss of weight at time of transplant. One point was assigned to each symptom, with the maximum score assigned being 5. A clinical score of 5 was also assigned to mice that died before the end of the 12-day period or were euthanized due to 30% loss of their initial weight. To assess significance, a 2way ANOVA was used with Bonferroni’s multiple comparisons test to determine if there was a significant difference in clinical scoring at each time point. The mean difference was calculated by determining the mean clinical score of all control (or experimental) mice at a particular time point (i.g. Mean 1 - Mean 2, at time point X). Researchers conducting the scoring were blinded to the identity of the recipients. Survival plots were generated by recording how many days a mouse survived post-transplantation with an end point of 12 weeks (84 days). Statistical significance of survival was determined using a Kaplan-Meier curve.

### 2.7 Mixed lymphocyte reaction

Fresh FoxP3-GFP splenocytes were harvested into complete RPMI media in the manner described previously. Before culture, cells were labeled with CellTrace™ Blue proliferation dye (Fisher Scientific, #C34574). 5 x 10^7^ cells were resuspended in 2.0 mL of PBS and stained with 2.5 μL of CellTrace stock solution for 20 min at 37 degrees C. The stain was performed according to manufacturer-provided protocol. The stained cells were then cultured for 6h in either vehicle (0.2%) or FV2 cocktail (3nM fluticasone propionate, 100nM venetoclax, and 20 ng/mL recombinant human IL-2) in complete RPMI. During culture time, BALB/c and FoxP3-GFP splenocytes were harvested and subjected to 2500 cGy of radiation. After 6h, cultured cells were harvested, washed in PBS, and plated in disclosed ratios with either irradiated BALB/c splenocytes (allogeneic stimulator cells) or irradiated FoxP3-GFP splenocytes (syngeneic stimulator cells). All ratios were calculated according to a scale with a baseline of 1.5 x 10^5^ cells. For example, the 5:5 ratio contained 7.5 x 10^5^ responder cells and 7.5 x 10^5^ stimulator cells. All MLR cultures were run for 72h at 37 °C.

### 2.8 Statistical analysis

All statistical tests were performed using GraphPad Prism 10. Statistical tests include (1) one-way ANOVA with Bonferroni’s multiple comparisons or Tukey’s multiple comparisons, (2) 2way ANOVA with Bonferroni’s multiple comparisons, (3) Mixed effects analysis with Bonferroni’s multiple comparisons, (4) Kaplan-Meier (simple survival analysis), (5) Paired T-test (6) Welch’s T-test and (7) Unpaired T-test. (* p < 0.03, ** p < 0.002, *** p < 0.001). Statistical test used is indicated in each figure legend.

## 3 Results

### 3.1 Combination of FLU and VEN increases regulatory T cell frequency *in vitro*

We first tested whether the combination of FLU and VEN could further decrease Tcon viability and increase the ratio of viable Tregs to Tcons (Treg: Tcon) during graft conditioning (**Figure 1**). To assess Treg percentage after cell conditioning we used a B6-FoxP3^EGFP^ (FoxP3-GFP) reporter mouse wherein all the Tregs fluoresce due to the insertion of GFP in the Treg specific FoxP3 (forkhead box protein P3) locus. We cultured 1 x 10^6^ splenocytes from a FoxP3^EGFP^ mouse for 18 hours in 3 nM FLU, or VEN in varying concentrations (1 nM, 10 nM, or 100 nM), or a combination of 3 nM FLU and VEN (1 nM, 10 nM, or 100 nM). We then measured the percentage of viable Tregs (out of viable CD4+ T cells) by flow cytometry as a surrogate for Treg: Tcon ratio (e.g. 50% viable Tregs = 1:1 ratio). We define viable cells as negative for propidium iodide (PI, marker of dead cells) and negative for annexin V (AnnxV, marker of apoptotic cells). We found that 100 nM VEN by itself had a modest but significant increase on the percentage of viable Tregs when compared to Veh, but lower than FLU alone (**Figure 1A**). Combining FLU with low dose VEN (1 nM) did not increase the Treg percentage (25.7% ± 8.2%) compared to FLU alone (22.3% ± 6.6%). However, the percentage of Tregs was significantly increased when combining FLU with either 10 nM VEN (56.0% ± 14.8%) or 100 nM VEN (56.0% ± 14.7%), compared to FLU alone. (**Figure 1A**; **Supplementary Figure 1A**).

**Figure 1:**
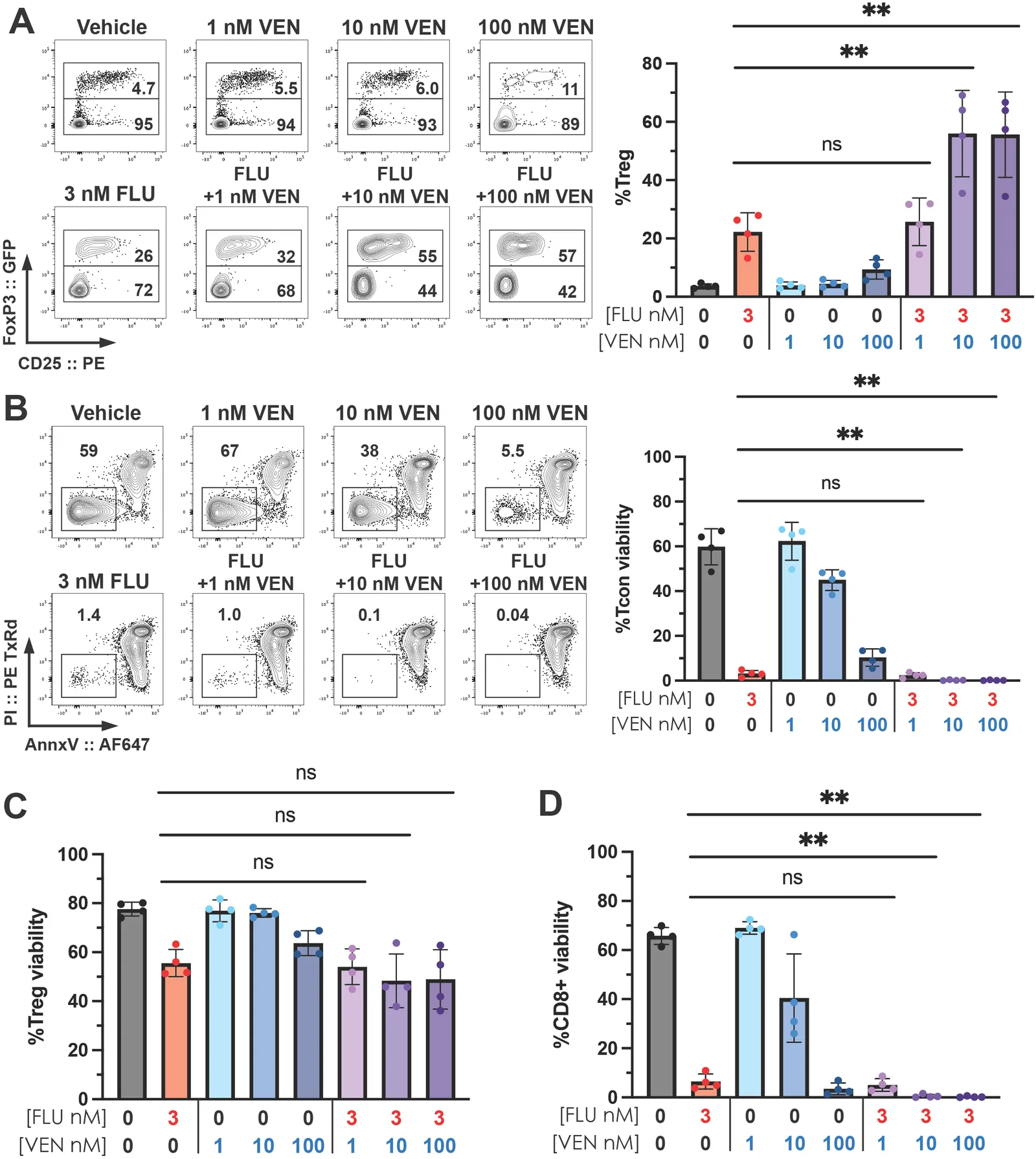
Effects of FLU and VEN on T cell viability during graft conditioning. **(A)** Comparison of percentages of viable (PI- Annexin V-) Tregs (FoxP3+) and viable CD4+ Tcons (FoxP3-) after 18h culture in Vehicle, FLU (3nM), VEN only (1, 10, 100 nM), and FLU (3nM) plus VEN (1, 10, 100 nM). Representative FACS plots are pregated on live, CD3+ CD4+ T cells. Bar graph (right) indicates the percentages of viable (PI- Annexin V-) Tregs out of total viable CD4+ T cells. **(B)** Viability (PI- Annexin V-) of Tcons after 18h culture in Vehicle (grey bar), FLU (3nM, red bar), VEN (1, 10, 100 nM, blue bars), or combinations of FLU + VEN (purple bars). Gates and bar graph (right) indicates the percentage of viable CD4+ Tcons out of total CD4+ Tcons. Tcons are gated as CD3+ CD4+ FoxP3-. **(C)** Bar graph of Treg viability (PI- Annexin V-) in Vehicle (grey bar), FLU only (red bar), VEN only (blue bars), or FLU + VEN (purple bars) culture. **(D)** Bar graph of CD8+ T cell viability (PI- Annexin V-) in Vehicle (grey bar), FLU only (red bar), VEN only (blue bars), or FLU + VEN (purple bars) culture. Statistical comparisons are between FLU +VEN conditions to FLU only. Error bars = SD, N = 4, one-way ANOVA with Bonferroni’s multiple comparisons, **p < 0.002, ns (not significant).

As the increase in Treg percentage after FLU culture is primarily due to a loss of Tcons, we also examined the impact of these drug combinations on Tcon viability after 18h graft conditioning (**Figure 1B**; **Supplementary Figure 1B**). We saw that there was no significant difference in Tcon viability (PI-, Annexin V-) from FLU + 1nM VEN compared to FLU alone. However, combining 3nM FLU with either 10 nM VEN (0.2% ± 0.2%) or with 100 nM VEN (0.2% ± 0.2%) both had significantly lower Tcon viability than with FLU alone (3.1% ± 1.4%). Moreover, Tcon viability in all FLU + VEN combination conditions was significantly lower than the vehicle control. Conversely, Tregs are more resistant to FLU-induced apoptosis compared to Tcons, and we found that the inclusion of VEN at any concentration in FLU culture did not significantly decrease Treg viability compared to FLU alone (**Figure 1C**; **Supplementary Figure 1C**). Lastly, we assessed CD8+ T cell viability and found viability decreases similar to that of Tcons, with no significant difference between FLU alone and FLU + 1 nM VEN conditioned cells. There was a significant decrease in CD8+ T cell viability when comparing FLU alone (6.5% ± 2.6%) to either FLU + 10 nM VEN (0.5% ± 0.7%) or FLU + 100 nM VEN (0.1% ± 0.3%) (**Figure 1D**; **Supplementary Figure 1D**). Thus, similar to our previous study of T cells in FLU culture, the combination of FLU + VEN has severe impacts on the viability of Tcons and CD8+ T cells, but little impact on Tregs. We conclude that the increase in Treg percentage in FLU + VEN culture is due primarily to the dramatic loss of Tcons.

### 3.2 Short term conditioning increases regulatory T cell frequency *in vitro*

A potential concern in trying to incorporate EVGC in the processing step of mobilized peripheral blood of donors, is that an 18 hour culture window may not be available to myeloablated patients awaiting transplantation. To address this, we determined whether reducing the amount of time required to condition cells could still achieve a high percentage of Tregs (**Figure 2**). We analyzed a variety of FLU and/or VEN combinations at three different time points (3, 6, and 9h) for their impact on the Treg: Tcon ratio. In cultures with FLU + 100 nM VEN we observed a significant increase in Treg frequency at 3, 6, and 9h compared to FLU alone. With FLU + 10 nM VEN we only observed a significant increase of Treg frequency at 9 hours (**Figure 2A**; **Supplementary Figure 2A**). We also examined Tcon viability and saw a significant decrease in Tcon viability at all three time points when comparing FLU alone to FLU + 100 nM VEN (**Figure 2B**; **Supplementary Figure 2B**). Importantly, we saw that Treg viability was intact at the 6h and 9h time points even in the harsher condition with FLU + 100 nM VEN (**Figure 2C**; **Supplementary Figure 2C**). CD8+ T cell viability was similar to Tcons (**Figure 2D**; **Supplementary Figure 2D**). Moving forward we decided to condition graft cells using 3 nM FLU + 100 nM VEN for 6h, due to a significant increase of Tregs from 7% to 31% and minimal impact to Treg viability. Thus, combining FLU and VEN allowed us to reduce the amount of culture time needed to increase the Treg: Tcon ratio from 18h down to 6h.

**Figure 2:**
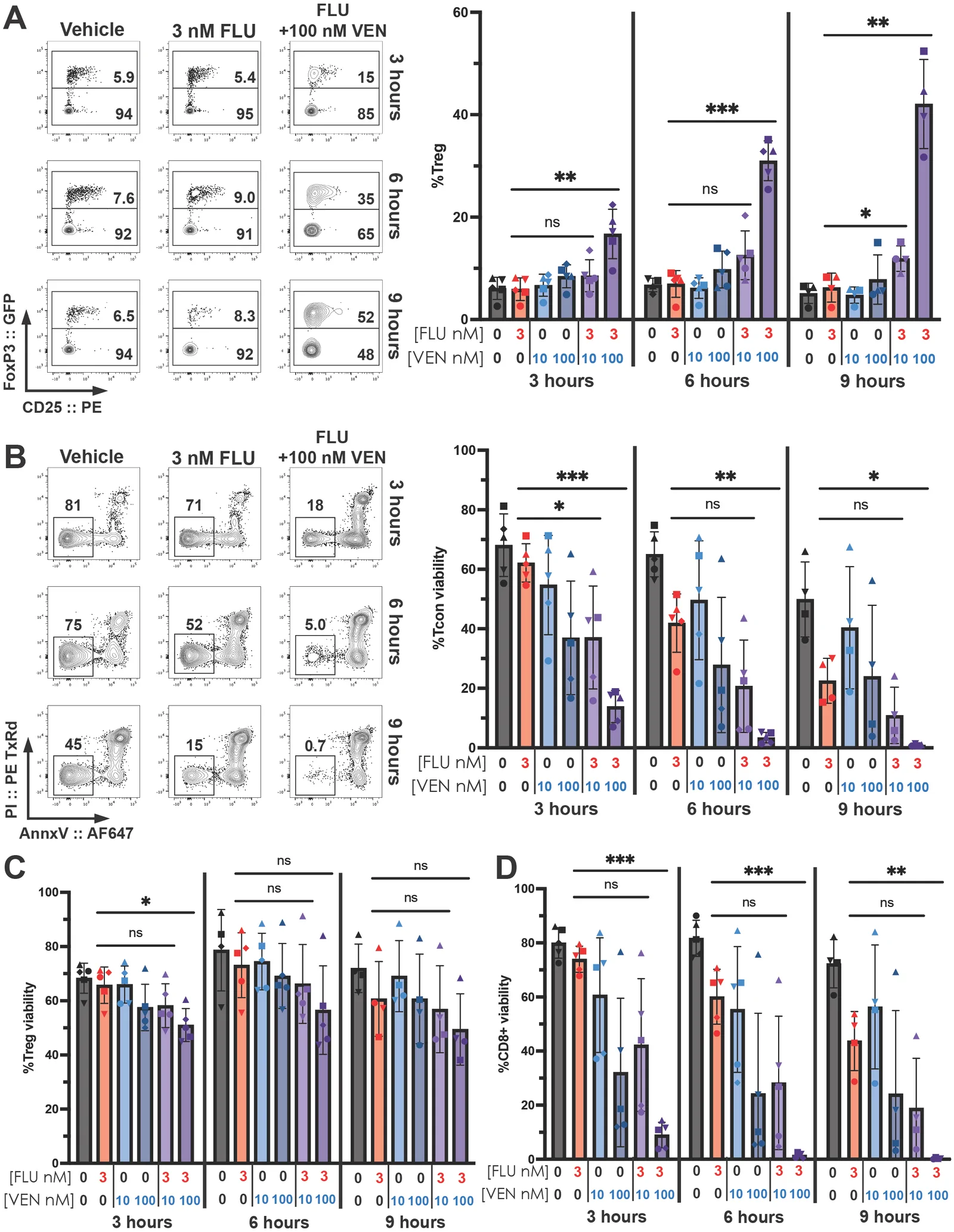
Short-term cultures of FLU with or without VEN on Treg: Tcon ratio and T cell viability. **(A)** FACS and bar graph analysis of viable Treg percentage (out of viable CD4+ T cells) after culture in Vehicle, FLU (3nM), VEN (10nM, 100nM), or FLU + VEN for 3, 6, and 9h. Representative FACS plots and gating of Tregs (FoxP3+) and Tcons (FoxP3-) at each time point is shown for three of the conditions, and is pregated on viable, CD3+ CD4+ T cells. Bar graphs show the percentage of viable Tregs in each condition. **(B)** FACS and bar graphs of Tcon viability after short-term culture. Gates and percentages of viable (PI- Annexin V-) Tcons (CD3+ CD4+ FoxP3-) are shown in each FACS plot for three representative conditions. Bar graphs show the percent of viable Tcons for all 6 conditions for 3 time points. **(C)** Bar graph of Treg (CD3+ CD4+ FoxP3+) viability (PI- Annexin V-) in all six conditions for all three timepoints. **(D)** Bar graph of CD8+ T cell (CD3+ CD8+) viability in all six conditions for all three timepoints. Data is a pool of 5 independent experiments, each experiment is denoted by a different symbol. Statistics: Error bars are SD (3h and 6h N = 5 and 9h N = 4), Mixed effects analysis with Bonferroni’s multiple comparisons test comparing FLU only to FLU + 10nM VEN (light purple) or FLU + 100nM VEN (dark purple), *p < 0.03, **p < 0.002, ***p < 0.001, ns (not significant).

### 3.3 Inclusion of the IL-2 cytokine with FLU and VEN further increases regulatory T cell frequency *in vitro*

Edinger et al., demonstrated that transplantation of Tregs and Tcons at a 1:1 ratio (i.e. 50% Tregs) reduced GVHD severity while preserving the beneficial GVL effect. We next tested whether the inclusion of IL-2 could further optimize our EVGC protocol to achieve this 1:1 ratio (**Figure 3**). We cultured FoxP3-GFP splenocytes for 6h exposing them to either vehicle, 3 nM FLU alone, or 3 nM FLU + 100 nM VEN and with or without 20 ng/mL recombinant human IL-2. We saw that the addition of IL-2 did not have an effect on Treg frequency in the vehicle or FLU only conditions, but we did observe a significant increase in Treg frequency in the FLU + VEN condition, increasing the relative percent of Tregs from 38.0% (± 4.0%) without IL-2 to 51.0% (± 1.3%) with IL-2 (**Figure 3A**). When comparing conditions with or without IL-2, no significant changes were observed in cell viability for Tcons, Tregs or CD8+ T cells (**Figures 3B-D** and **Supplementary Figure 3A**). Despite no difference in Treg viability with the inclusion of IL-2, the relative quantity of Tregs in the FLU + 100 nM VEN condition increases with IL-2 compared to without IL-2 (**Figure 3E**). While IL-2 can induce Treg proliferation, in the short 6h culture time we interpret these results as IL-2 providing a signal to maintain FoxP3 expression and/or preserve viability during EVGC (15).

**Figure 3:**
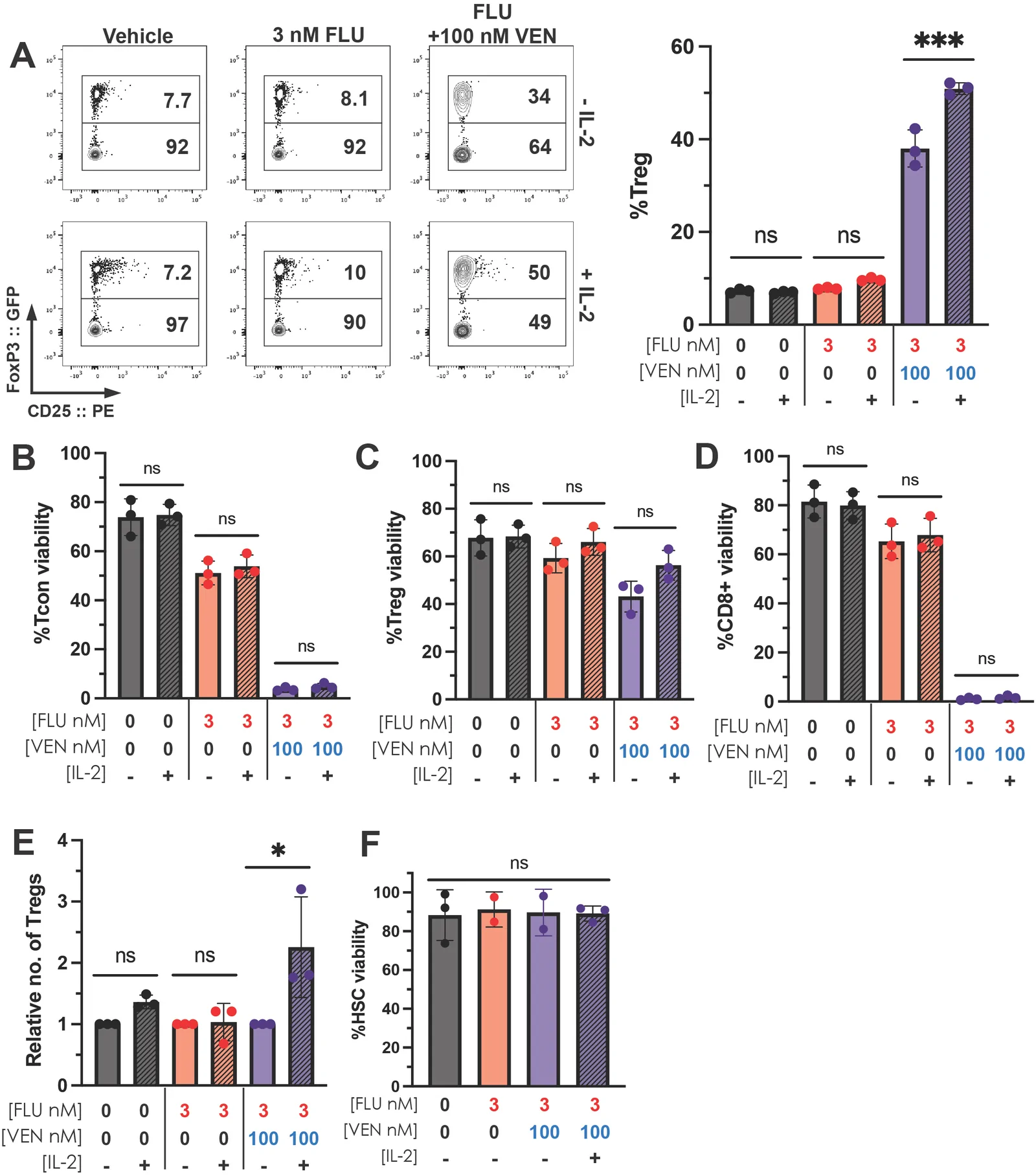
Effects of IL-2 on T cell and HSC viability in the presence of FLU and VEN. **(A)** Representative FACS plots and bar graph analysis of the percentage of viable Tregs (FoxP3+), and Tcons (FoxP3-) out of total viable CD4+ T cells without (top row), or with (bottom row) the addition of 20 ng/mL recombinant human IL-2 (rhIL-2). Bar graph on the right indicates percent of viable Tregs without (solid bars) or with IL-2 (striped bars) for Vehicle (grey), 3nM FLU (red), or 3nM FLU + 100nM VEN (purple). **(B-D)** Comparison of the viability of Tcons (CD4+ FoxP3-), Tregs (CD4+ FoxP3+) and CD8+ T cells in Vehicle, FLU, or FLU + VEN with or without IL-2. **(E)** Relative number of viable Tregs were calculated by taking the absolute number of viable Tregs (per million cells) in the condition with IL-2, divided by the absolute number of viable Tregs in the condition without IL-2. Absolute numbers were calculated using reference beads for each condition. **(F)** Comparison of BM HSC viability in Veh, FLU, FLU + VEN, and FLU + VEN + IL-2 after 6h culture. Statistics: **(A-E)** N = 3, one-way ANOVA with Tukey’s multiple comparison, **(F)** N = 3, one-way ANOVA,.

HSC viability is critical to the success of HCT. To ensure there was no impact on the viability of HSCs, we cultured BM cells with vehicle, FLU alone, and FLU + 100 nM VEN with or without IL-2 and found no differences in HSC viability (**Figure 3F** and **Supplementary Figure 3B**). This suggests that culturing donor grafts in the triple drug combination will not detrimentally affect HSC function or HCT success.

As glucocorticoids like FLU are immunosuppressive, we next assessed the ability of T cells that survive graft conditioning to become activated *in vitro*, which could be important for maintaining the potential for GVL. We conditioned spleen cells in vehicle, FLU alone, or FLU + VEN + IL-2 for 6h, then washed the cells and activated them using agonist antibodies to CD3 and CD28 for 5 days. We then measured the frequency of T cells expressing the activation markers PD-1 and CD25, and found no significant differences in the ability of Tcons or CD8+ T cells to upregulate both markers, indicating the T cells that survive EVGC are capable of being activated in a strong activating condition (**Supplementary Figures 4A, B**). Due to the powerful activation response that occurs with CD3/CD28 stimulation, we also performed a mixed lymphocyte reaction (MLR), which may better mimic allogeneic activation. We cultured B6 FoxP3-GFP cells labeled with CellTrace Blue proliferation dye in either vehicle or FLU + VEN + IL-2 for 6h, and then mixed them with irradiated BALB/c splenocytes for 3 days. We found that vehicle conditioned cells had a significantly higher percentage of activated Tcons and CD8 T cells compared to FLU + VEN + IL-2 conditioned cells at varying ratios (5:5, 5:2, 10:3, and 10:2 of FoxP3:BALB/c) (**Supplementary Figures 4C–F**). This suggests that while T cells that survive FV2 culture are capable of being activated by a strong activation signal (anti-CD3/CD28 antibodies), they have a diminished response to an APC-driven allogeneic activation condition (MLR), which has implications for both GVHD and GVL.

Taken together, these data demonstrate that conditioning donor grafts in 3 nM FLU, 100 nM VEN, and 20 ng/mL IL-2 for 6h significantly increases the ratio of Treg: Tcon to nearly 1:1, without affecting HSC viability. Thus, we chose this conditioning regimen, which we term “FV2”, when performing our *in vivo* studies assessing GVHD and survival.

### 3.4 *Ex vivo* graft conditioning in FLU, VEN, and IL-2 reduces GVHD severity and increases overall survival

To test the efficacy of EVGC to reduce GVHD *in vivo*, we used a fully MHC-mismatched allogeneic transplant model: B6 FoxP3-GFP (H-2^b^) whole bone marrow and splenocytes transplanted into BALB/c (H-2^d^) recipients. We first conditioned 5 x 10^6^ FoxP3-GFP splenocytes in either vehicle (DMSO) or 3 nM FLU with 100 nM VEN and 20 ng/mL IL-2 (FV2 condition) for 6h. We then transplanted the 5 x 10^6^ conditioned splenocytes along with 1 x 10^6^ unconditioned whole bone marrow cells (also FoxP3-GFP) into lethally-irradiated BALB/c recipients (**Figure 4A**). We then tracked both GVHD clinical severity and overall survival. We assessed the clinical severity of GVHD in the recipients using the grading rubric described in Naserian et al. (16). In a blinded study, recipient mice were graded for weight loss, ruffled fur, hunched back, diarrhea and skin lesions, assigning one point for each observed symptom. We graded mice for the first 12 days post-transplantation and found that recipients receiving FV2 treated cells had significantly lower clinical scores and visibly reduced symptoms by the end of the scoring period compared to recipients receiving vehicle treated cells (**Figures 4B, C**). Moreover, in a pool of four independent experiments we found that mice receiving FV2 conditioned cells had significantly higher overall survival compared to mice receiving vehicle-conditioned grafts (**Figure 4D**). While all recipients receiving vehicle-conditioned grafts died (16/16 died), only 22% of recipients receiving FV2-conditioned grafts died (4/18 died). This is approximately half the death we observed in our previous study examining FLU-conditioned grafts, where half the recipients died of GVHD (12). Thus, conditioning fully-mismatched grafts in the combination of FLU, VEN, and IL-2 for 6h dramatically reduced the incidence and severity of GVHD after allogeneic transplantation, increasing survival from 0% in the control to over 75% in the FV2 group.

**Figure 4:**
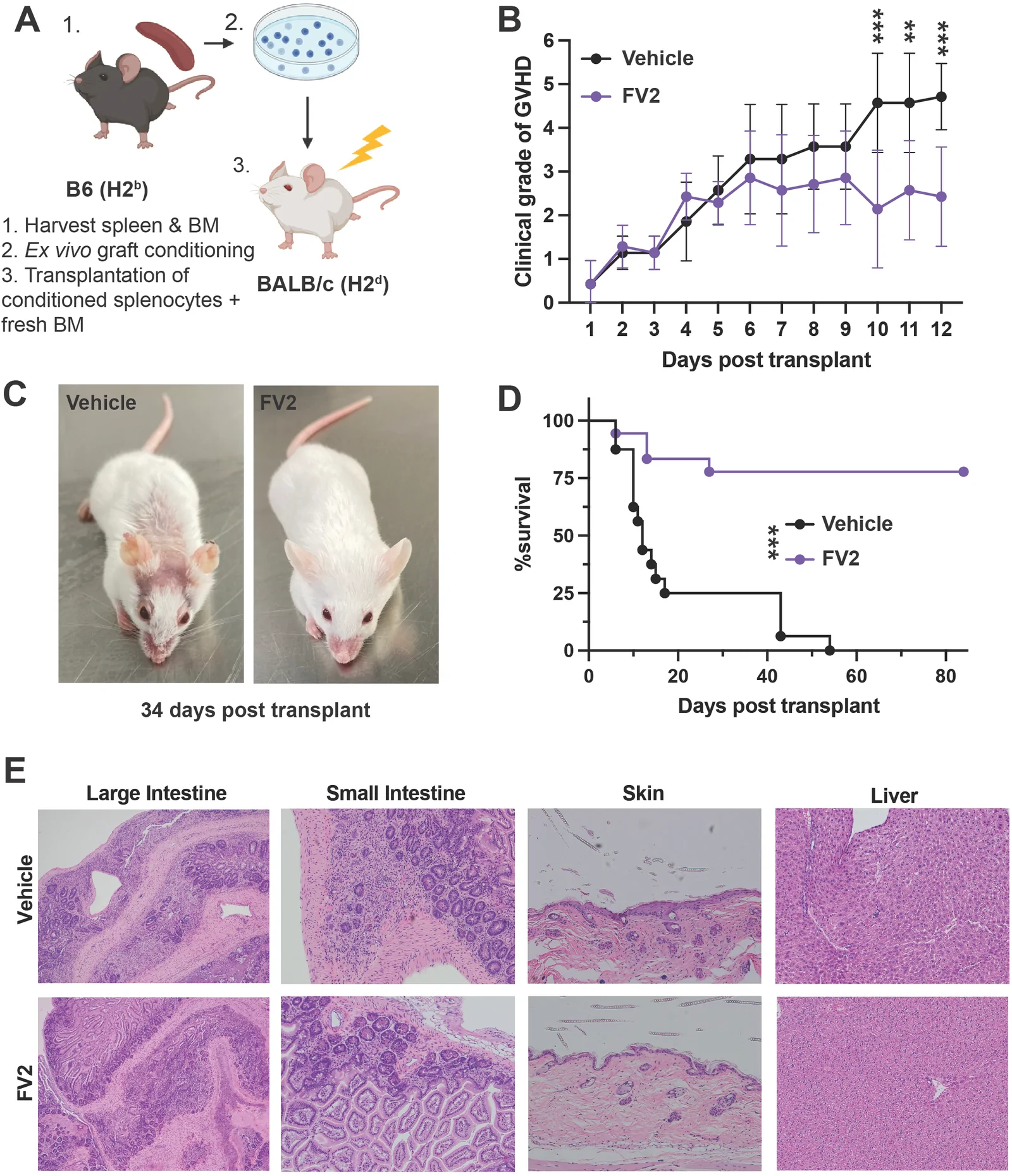
*In vivo* analysis of graft conditioning effects with FLU + VEN + IL-2 on GVHD after allogeneic transplant. **(A)** Transplantation strategy. B6 (H-2^b^) spleen cells are conditioned for 6h in Vehicle or 3nM FLU + 100nM VEN + 20ng/mL IL-2 (“FV2”), then transplanted into irradiated BALB/c (H-2^d^) recipients. **(B)** Measurement of clinical grade of recipients given Vehicle-treated cells (black line, N = 16) or FV2-treated cells (purple line, N = 18) for 12 days (pooled from 4 independent experiments). Clinical score based on visual inspection of recipients with 1 point given for each GVHD symptom (weight loss > 10%, hunched back, ruffled fur, diarrhea, and hair loss). **(C)** Representative images of recipients of Vehicle-treated (left) and FV2-treated (right) grafts, 34 days after transplantation. **(D)** Survival curve of recipients given Vehicle-treated (black, N = 16) or FV2-treated (purple, N = 18) grafts (pooled from 4 independent experiments). **(E)** Representative images for H&E stained tissues 10–12 days post-transplantation. Large intestines are imaged at 4X, small intestine, liver, and skin are imaged at 10X. Statistics: **(B)** 2way ANOVA with Bonferroni’s multiple comparisons test at each timepoint, **(D)** Kaplan-Meier (simple survival analysis). ***p < 0.001.

To examine GVHD target tissue damage after allogeneic transplantation, we harvested large intestine, small intestine, liver and skin samples 10–12 days post-transplantation. These tissues were submitted to the UC Irvine pathology core and were sectioned and stained with hematoxylin and eosin. In a blinded study, the pathologist provided descriptions of various prepared tissue samples. The pathologist noted that the large intestine of the vehicle recipient mice had distinct areas of lymphocytic infiltration whereas mice receiving FV2 treated cells had little to no infiltration. We observed minor increases in intraepithelial lymphocytes within the small intestines of vehicle mice which was not observed in FV2 mice. Lastly, there were no remarkable differences in the liver or skin tissue section of either vehicle or FV2 mice (**Figure 4E**). Notably, these effects were achieved without directly injecting any of the compounds into recipient mice, but rather only by pretreating the grafts with these compounds, thus avoiding any potential side effects that these immunosuppressive drugs may cause to immunocompromised patients.

### 3.5 *Ex vivo* graft conditioning in FV2 reduces GVHD in haploidentical transplant model

Due to the increasing prevalence of haploidentical transplants to treat blood malignancies, we sought to test our EVGC protocol in a half-matched mouse model of GVHD. We developed a haploidentical transplant system by first crossing a B6 FoxP3-GFP (H-2^b^) mouse with a C3H (H-2^k^) which we termed “FoxC” (H-2^bk^) to serve as the donor strain. For the recipient, we crossed a B6 CFP+ (H-2^b^) mouse with a BALB/c (H-2^d^) mouse, which we termed “Hy5” (H-2^bd^) (**Figure 5A**). Thus, the donors and recipients will share half their H-2 alleles with the B6 (H-2^b^) background, but differ in their second alleles (H-2^k^ v. H-2^d^). We assessed donor chimerism by frequency of CFP- cells. To determine the efficacy of EVGC in our haploidentical transplant, we cultured the spleen cells from a FoxC donor for 18h in 3 nM FLU, 10 nM VEN and IL-2 (BM stored at 4 °C until transplant) and confirmed the increased Treg percentage in the FV2 condition prior to transplantation (**Supplementary Figure 5A**). After EVGC, conditioned splenocytes and unconditioned BM were transplanted into lethally-irradiated 1050 cGy) Hy5 recipient mice (**Figure 5B**). We then tracked percent weight change as a proxy for GVHD severity and observed that Hy5 mice receiving FV2-treated cells regained weight similar to the BM control and mice receiving vehicle treated cells had sustained significant weight loss (**Figure 5C**). Moreover, the physical appearance of FV2-recipents was much improved compared to the vehicle control (**Figure 5D**). Additionally, the overall survival of FV2-recipients was significantly higher than the vehicle-recipients after the 12 week post-transplant period (**Figure 5E**). While no vehicle recipients survived to 12 weeks, we assessed chimerism of FV2 recipients at 12 weeks post transplant by measuring donor derived granulocytes in the spleen, of which 90% were donor-derived, indicating robust donor engraftment (**Supplementary Figure 5B**). As granulocytes are the shortest lived hematopoietic cell type in the blood, their chimerism is considered most reflective of HSC chimerism in the BM. Taken together, this data demonstrates that EVGC can also be used in a haplo-identical transplant model to reduce GVHD and increase survival.

**Figure 5:**
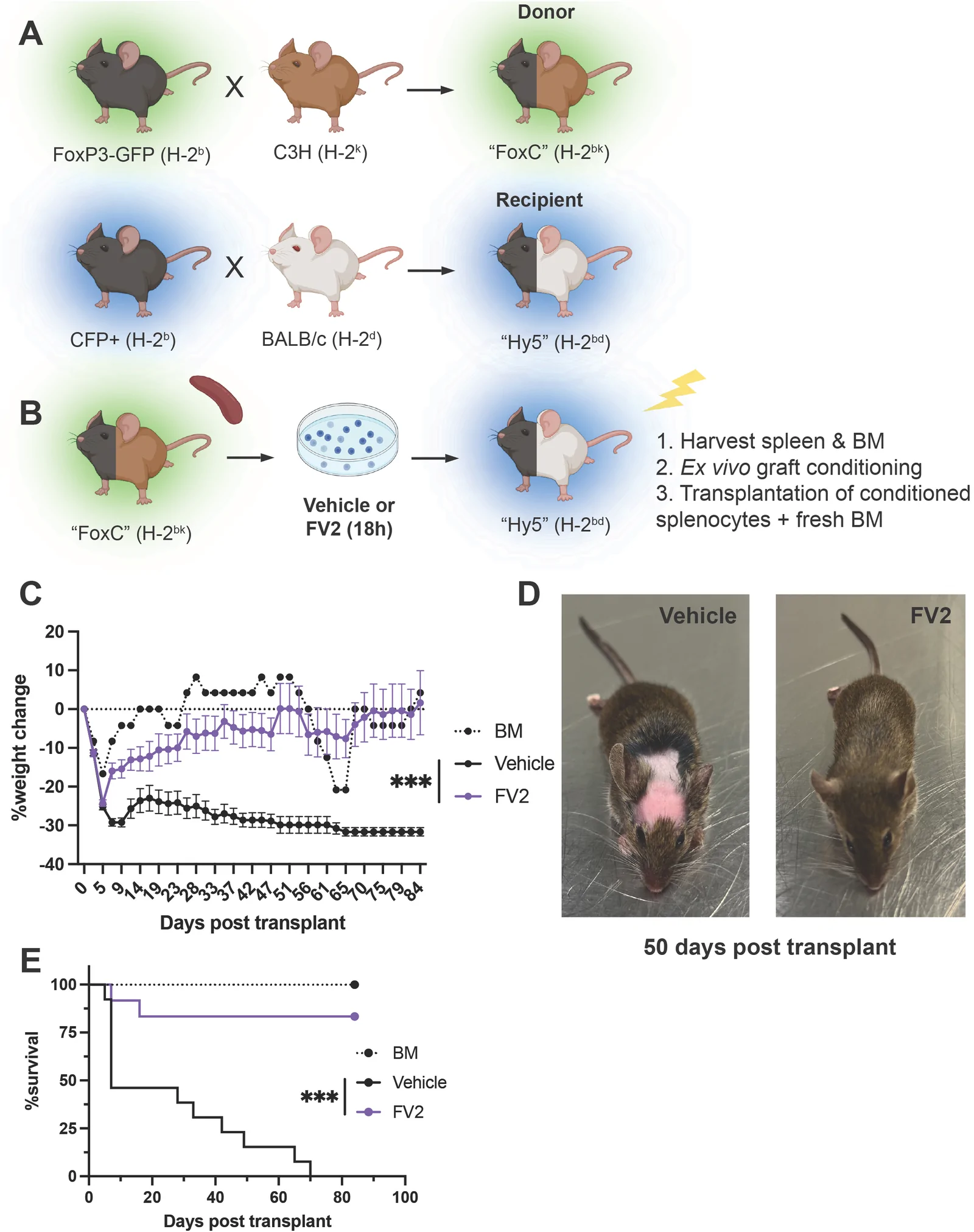
*In vivo* analysis of graft conditioning on GVHD in haploidentical transplantation. **(A)** Breeding scheme to generate haploidentical strains. B6 FoxP3-GFP (H-2^b^) mice are bred to C3H (H-2^k^) mice to generate “FoxC” (H-2^bk^) donors (top row). CFP+ B6 (H-2^b^) are bred to BALB/c (H-2^d^) to generate Hy5 (H-2^bd^) recipients (bottom row). **(B)** Experimental Strategy. Donor “FoxC” splenocytes are cultured for 18h in vehicle or FV2 (3nM FLU, 10nM VEN, 20 ng/mL IL-2) and transplanted along with fresh BM cells to lethally irradiated “Hy5” recipients. **(C)** Percent weight change of BM only (black dotted line, N = 1), vehicle (black solid line, N = 13), or FV2 (purple line, N = 12) recipients, measured three times a week for 12 weeks post transplant. **(D)** Representative images of mice receiving either vehicle (left) or FV2 (right) conditioned cells 50 days post-transplant. **(E)** Overall survival of mice receiving either BM only (black dotted line, N = 1), vehicle (black solid line, N = 13), or FV2 (purple line, N = 12) conditioned cells. (Pooled from 2 separate experiments). Statistics: **(C)** 2way ANOVA (Mixed effects analysis) with Bonferroni’s multiple comparisons. **(E)** Kaplan-Meier (simple survival analysis). ***p < 0.001.

### 3.6 Mice receiving EVGC cells have reduced inflammatory response following allogeneic transplantation

To better understand the donor T cell effector response following allo-HCT, we analyzed the ratios of effector T cell subsets in mice that received either vehicle or FV2-treated cells (**Figure 6**). To do this, we transplanted 1 x 10^6^ unconditioned BM cells with 5 x 10^6^ conditioned cells from B6 FoxP3-GFP donors into lethally-irradiated BALB/c recipients. On day 10 post-transplantation, we collected spleens and incubated the cells with PMA/ionomycin to stimulate cytokine production, and monensin/brefeldin A to block cytokine secretion, then analyzed cytokine production via intracellular flow cytometry (**Figure 6A**). We identified Th1, Th2, Th17, and Treg cells based on their cytokine secretion of IFN*γ* (Th1), IL-4 (Th2), or IL-17 (Th17), and FoxP3 expression (Treg). Here, we found that mice receiving FV2-conditioned cells had significantly lower Th1 (CD4+ IFN*γ*+) effector cells (32.0% ± 24.9%) compared to the vehicle control (80.1% ± 6.3%), but no difference in IFN*γ* expression in the CD8+ compartment (**Figure 6B** and **Supplementary Figure 6A**). Th2 (CD4+ IL-4+) effector cells were not significantly different between the two groups, but Th17 (CD4+ IL-17+) effector cells were significantly lower in the FV2 group (**Figures 6C, D**). Interestingly, there was an increased percentage of CD8+ IL-4+ cells in the FV2 group (**Figure 6C**). We wanted to determine if the increased frequency of Tregs observed *in vitro* after FV2-conditioning was maintained *in vivo.* In the FV2 group, we saw a significant increase in donor Treg percentage within the spleen 10 days post transplant, with over 50% Treg frequency, indicating the increased Treg: Tcon ratio created by the FV2 culture was maintained *in vivo* even 10 days after the cells were removed from the FV2 cocktail (**Figure 6E**; **Supplementary Figure 6B**). Lastly, we wanted to confirm that the differences in GVHD development between the two groups was not solely due to loss of T cells following EVGC. Donor-derived T cells made up approximately 40-50% of all donor-derived cells in either Veh or FV2 recipients, and were not significantly different between the two groups, indicating an expansion of T cells in both groups immediately following allogeneic transplantation (**Figure 6F**). We also compared the absolute numbers of Tcons, CD8+ T cells, and Tregs at the end of conditioning (pre-transplant) compared to 10 days post-transplant in the spleen (**Figure 6G**). For Tcons and CD8+ T cells, there was a large, approximately 12-fold decrease in the absolute number of live cells between vehicle and FV2 treatments pre-transplant. However, 10 days post-transplant, the FV2-treated T cells began to catch up, and were only about 4-fold lower compared to vehicle-treated T cells. Absolute numbers of Tregs pre-transplant and 10 days post-transplant were not significantly different in either condition. These data indicate that while FV2-conditioned grafts initially had much lower Tcons and CD8+ T cells at the time of transplant compared to vehicle-conditioned grafts, they were still capable of allogeneic activation and expansion, and even began to catch up to their vehicle counterparts, albeit at a reduced level. Thus, both groups given either vehicle or FV2 treated donor cells displayed donor T cell expansion, but the donor T cells from the FV2 condition were significantly less activated. This is consistent with our previous study using FLU alone and suggests allogeneic T cell expansion without activation in the FV2 condition.

**Figure 6:**
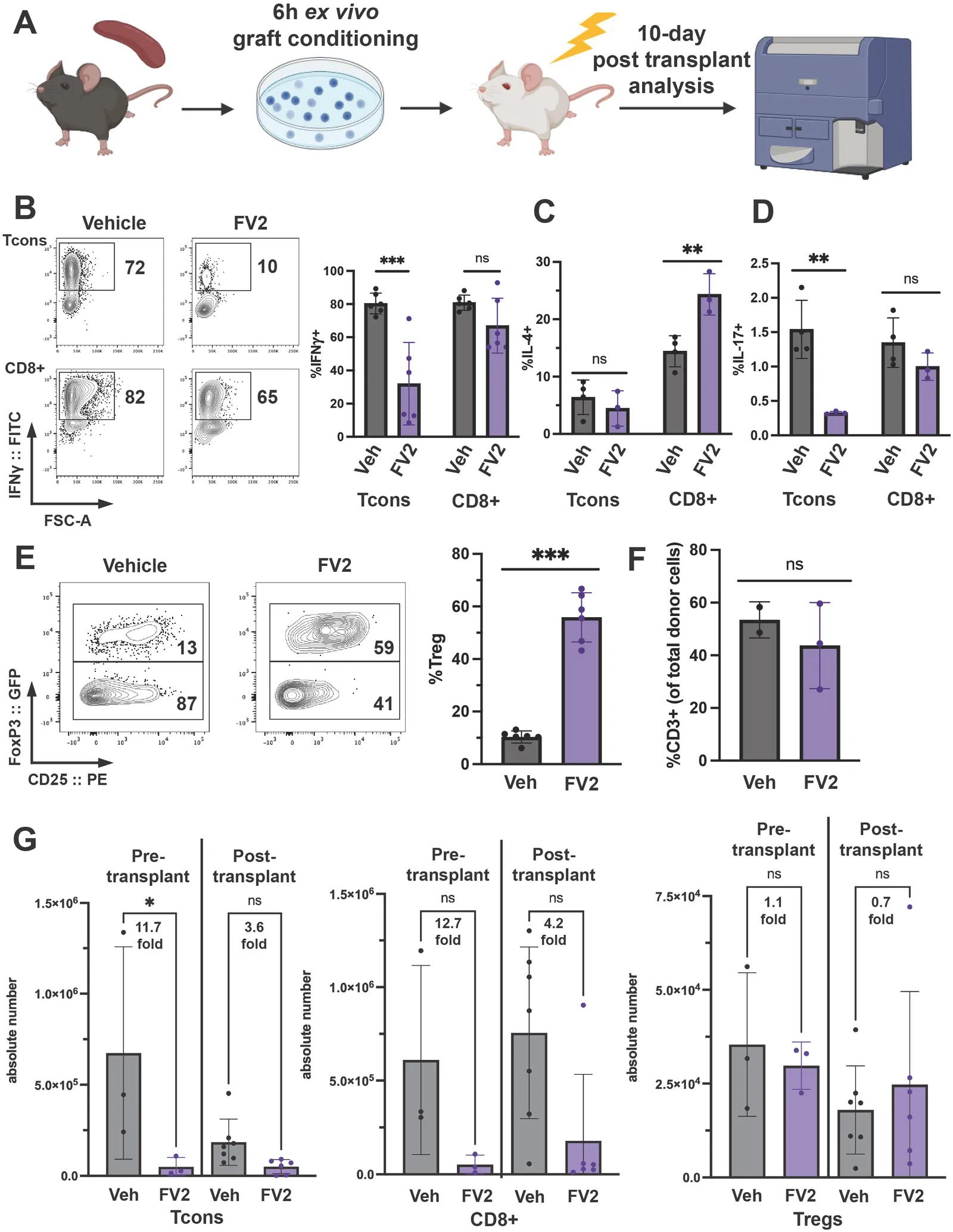
Analysis of effector T cell subsets 10 days after allogeneic transplantation with Vehicle- or FV2-conditioned grafts. **(A)** Experimental Strategy. B6 spleens were conditioning for 6h in Vehicle or FV2, then transplanted with fresh B6 BM into irradiated BALB/c recipients. Spleens were harvested from recipients 10 days post-transplantation, then stimulated for 4h to induce cytokine secretion, then intracellularly stained and analyzed by FACS. **(B)** Representative FACS plots of IFN*γ* secretion in stimulated Tcons (FoxP3-, top row) or CD8+ T cells (bottom row) of recipient spleens given vehicle-treated (left) or FV2-treated (right) grafts. Gates and percentages of IFN*γ*+ T cells is shown. Bar graphs showing IFN*γ*+ Tcons (Th1) and CD8+ T cells from Vehicle (grey) and FV2-treated (purple) grafts. **(C)** Percentage of IL-4+ Tcons (Th2) and CD8+ T cells. **(D)** Percentage of IL-17+ Tcons (Th17) and CD8+ T cells. **(E)** FACS plots and bar graph showing the percentage of Tregs (FoxP3+) and Tcons (FoxP3-) in unstimulated spleen cells from recipients given Vehicle- or FV2-treated grafts. **(F)** Bar graph showing the percentage of donor (H-2^b^+ H-2^d^-) T cells (CD3+) out of total donor (H-2^b^) cells. **(G)** Bar graphs showing absolute number of live Tcons, CD8+ T cells, and Tregs, after conditioning (pre-transplant) and 10 days post-transplant. Pre-transplant numbers represent the amount of each cell type transplanted per recipient mouse. Statistics: **(B-D)** 2way ANOVA with Bonferroni’s multiple comparison test, **(B)** N = 6, **(C, D)** N = 4 (Veh), N = 3 (FV2), error bars are SD, pool of 2 independent experiments, **(E)** unpaired T-test, N = 6, **(F)** unpaired T-test, N = 2 (Veh), 3 (FV2). **(G)** one-way ANOVA with Tukey’s multiple comparison test. *p < 0.033, **p < 0.002, ***p < 0.001, ns (not significant).

## 4 Discussion

We previously demonstrated that pre-conditioning donor cells with FLU alone prior to fully allogeneic transplantation reduced GVHD severity and increased overall survival from 0% to 50% up to 120 days post-transplantation (12). In this study, we conditioned grafts for 6h in a triple combination of 3 nM FLU + 100 nM VEN + 20 ng/mL IL-2 (FV2) and further improved the overall survival to over 75% for 12 weeks post-transplant, in both fully mismatched and the more clinically relevant haploidentical transplantation models (**Figure 4D**, **5E**). While Tcon and CD8+ T cell viability were dramatically reduced after FV2 conditioning, 10 days after transplantation those T cell subsets had expanded and partially recovered in the recipients (**Figures 3B, D**, **6F, G**). However, T cells in mice receiving FV2-conditioned grafts displayed lower expression of activation markers, a lower fraction of Th1 effector T cells, and an increase in the proportion of Tregs (**Figures 5B, E**). Our interpretation of these results is that GVHD was reduced with FV2-conditioned grafts due to the increase of the Treg: Tcon ratio in the grafts after the 6h culture. However, the reduced number of Tcon and CD8 T cells in the FV2 recipients (approximately 4-fold lower at day 10) may also be a contributing factor in the reduced GVHD. As the Treg: Tcon ratio is maintained after transplantation, this suggests both populations of T cells become allogeneically activated, but the expanded Tregs are able to suppress the expanded Tcons from triggering a GVHD response. Tregs are well established as important for inducing tolerance upon transplantation and can reduce GVHD while permitting GVL if transplanted at a 1:1 ratio with Tcons (11). However, those ratios were generated using a cell sorting approach, which poses a technical challenge when trying to implement this in a clinical setting, primarily, the ability to sort cells under sterile conditions with high efficiency.

Our model for how these three compounds synergize to increase the Treg: Tcon ratio is as follows: First, treatment of T cells with glucocorticoids leads to upregulation of Bim (*Bcl2l11*), a pro-apoptotic BH3-only member of the Bcl2 family (17). While the *Bcl2l11* promoter does not contain a glucocorticoid response element, GCs can indirectly induce Bim expression by transactivation of *Foxo3a*, which transactivates Bim (18), or by repression of miRNAs that target the Bim 3’UTR (19), or by depleting ATP which causes AMPK activation of Foxo3a (20). Bim induces apoptosis by blocking the anti-apoptotic function of Bcl2 but can also promote the pro-apoptotic function of BAX and BAK (21). In our previous study, we showed that GCs alone are sufficient to induce apoptosis in T cells (12), and here we find that the addition of the Bcl2 inhibitor Venetoclax blocks Bcl2 activity and accelerates the process, increasing Tcon and CD8 T cell apoptosis and further increasing the ratio of Tregs: Tcons. Second, Tregs are resistant to GC-induced apoptosis (22), likely due to their greater reliance on Mcl1, another anti-apoptotic Bcl2 family member (23). Tregs reliance on Mcl1 over Bcl2 protects them from the effects of venetoclax. Third, IL-2 signaling induces STAT5-dependent transcription to maintain high FoxP3 expression during culture (24, 25), and also maintains high Mcl1 expression in Tregs (23).

Prior research by us and others demonstrated the potential for conditioning HSCs in Fluticasone Propionate (FLU) to enhance engraftment by upregulating CXCR4, the bone marrow homing receptor (12, 26). However, possible long-term effects of HSC exposure to venetoclax during graft conditioning are unknown. Venetoclax is FDA approved for treatment of acute myeloid leukemia (AML) and chronic lymphocytic leukemia (CLL). A recent clinical study observed that AML patients who received venetoclax (in combination with either hypomethylating agents or low dose cytarabine) had significantly higher overall survival and higher complete remission rates compared to patients receiving conventional chemotherapy. Importantly, venetoclax recipients had significantly lower rates of neutropenia and thrombocytopenia compared to chemotherapy patients, demonstrating that venetoclax administration did not worsen negative side effects that are associated with chemotherapy (27). A comprehensive retrospective study of multiple randomized clinical trials determined that in the context of CLL, venetoclax did not pose additional risk of infectious adverse effects. They noted that there was not enough evidence to determine whether venetoclax posed additional risks in AML patients, but addressed that there were likely higher rates of high grade neutropenia. Taken together, it is reasonable to infer that venetoclax does not pose a higher risk to hematopoiesis and white blood cell function compared to conventional chemotherapy in AML and CLL patients (28).

A potential caveat to EVGC is that conditioning grafts with powerful immunosuppressive and/or tolerance-inducing compounds may reduce the effectiveness of donor immune cells to mediate an anti-cancer GVT/GVL response, a critical component of allogeneic-HCTs. However, there are several pieces of evidence that suggest that graft conditioning may still permit a robust GVL response. First, T cells that survive graft conditioning are capable of being activated with CD3/CD28 antibodies (**Figure 3G**). Second, FV2-conditioned T cells expand rapidly following allogeneic transplant and fill the donor hematopoietic pool to nearly the same extent as vehicle-treated grafts (**Figure 6F**). Third, CD8+ T cells from recipients receiving FV2-conditioned grafts can be stimulated to produce IFN*γ* at the same level as recipients receiving vehicle-conditioned grafts (**Figure 6B**). This implies the retention of some functionality in the CD8+ T cell compartment, which is critical for preserving the beneficial GVT effect of allo-HCT. Finally, while the increased frequency of Tregs might imply a reduction in GVT response, prior research demonstrated that allogeneic Tregs home to secondary lymphoid tissues and sites of GVHD where they suppress Tcon activation, but do not inhibit the GVT response at tumor sites (29). Conversely, the overall reduction in both Tcons and CD8+ T cells in the FV2-treated grafts may have negative effects on GVT activity, as prior research has shown a minimum threshold of T cells needed to drive GVT, a threshold the FV2 conditioned grafts are unlikely to reach (11). Furthermore, the reduced allogeneic activation of FV2 treated T cells in the MLR (**Supplementary Figure 4**) suggests the treatment may cause some reduction in T cell activation potential that could dampen a GVT response, even amongst the T cells that survive conditioning. Future experiments will be required to determine if EVGC with the FV2 combination affects the ability of T cells to mount an effective anti-tumor response, and/or whether additional compounds are needed to preserve the GVT activity while still suppressing GVHD. Even if EVGC proves unusable for allo-HCT treatments for blood cancers, it could still be valuable in treating hematologic disorders that do not require a GVT response, such as severe aplastic anemia, sickle cell disease, and thalassemia major.

The key advantage that EVGC offers over other graft manipulation approaches, is that it does not require cell sorting or T cell expansion which can be costly, time consuming, and challenging to implement in clinical settings. A GMP facility is required for both strategies. If EVGC could be performed in the apheresis collection bags containing donor HCT grafts, it could be done in a stem cell processing center, where grafts are normally received and processed prior to delivery to hospitals for transplantation. This would avoid the need for a GMP facility, saving both time and cost. Moreover, an exciting feature of EVGC in this study is that no mice were given any drugs but rather the observed effect was solely due to EVGC. As post-transplant cyclophosphamide (PTCy) is now the gold standard for GVHD prophylaxis, we see an opportunity to combine PTCy with EVGC to potentially reduce the amount of toxic Cy or other immunosuppressive compounds administered to patients and reduce treatment related mortality or other adverse events. Future studies will be needed to test whether a combination of EVGC with reduced dose PTCy can be more effective at preventing GVHD than PTCy alone.

## Data Availability

The raw data supporting the conclusions of this article will be made available by the authors, without undue reservation.
