# Supplementary material for: Short term ex vivo graft conditioning with venetoclax, fluticasone propionate, and IL-2 reduces graft versus host disease in mouse allogeneic hematopoietic cell transplantation
Source: Front Immunol. 2026 Sep 24;17:1746010. doi: 10.3389/fimmu.2026.1746010 (PMC13645692; doi:10.3389/fimmu.2026.1746010)
Supplement: Supplementary file 1 [file SupplementaryFile1.docx]

**SUPPLEMENTARY MATERIAL**

Short Term Ex Vivo Graft Conditioning with Venetoclax, Fluticasone Propionate, and IL-2 Reduces Graft Versus Host Disease in Mouse Allogeneic Hematopoietic Cell Transplantation

L Angel Ayala^1,2^, Alizeh Ahmedani^1,2^,Cuiwen Zhou^1,2^, Gabriella L Hisoire^1,2^, Alexii Villamar^1,2^, Michelle Sandra Baez^1,2^, Nathalia Onofre^1,2^, Chanel De Smet^1,2^, Pauline U Nguyen^2^, Vanessa M Scarfone^2^, Erika S Varady^1,2^, Xiyu Chen^1,2^, Ankita Daud^1,2^, and Matthew A Inlay^1,2,^*.

^1^Department of Molecular Biology and Biochemistry, University of California Irvine, Irvine, CA, USA

^2^Sue and Bill Gross Hall Stem Cell Research Center, University of California Irvine, Irvine, CA, USA

***Correspondence:**

Matthew Inlay

minlay@uci.edu

**SUPPLEMENTARY TABLE 1**

**Antibodies for flow cytometry**

| **Antigen** | **Clone** | **Conjugate** | **Source** | **Catalog** |
| --- | --- | --- | --- | --- |
| CD3ε | 500A2 | Alexa Fluor 700 | BioLegend | 152316 |
|  | 145-2C11 | PE/Cyanine5 | BioLegend | 100309 |
|  | 17A2 | PE/Cyanine7 | BioLegend | 100220 |
| CD4 | RM4-5 | BV605 | BioLegend | 100547 |
|  | RM4-5 | PE/Cyanine7 | BioLegend | 100528 |
| CD8a | 53-6.7 | APC/Cyanine7 | BioLegend | 100713 |
|  | 53-6.7 | PE/Cyanine7 | BioLegend | 100722 |
| CD19 | 6D5 | BV421 | BioLegend | 115537 |
| CD25 | PC61 | PE | BioLegend | 102007 |
|  | PC61 | APC | BioLegend | 102011 |
| CD27 | LG.3A10 | APC/Cyanine7 | BioLegend | 124226 |
| CD34 | RAM34 | FITC | BD Biosciences | 560238 |
| CD45 | 30-F11 | BV785 | BioLegend | 103149 |
| Annexin V |  | Alexa Fluor 647 | BioLegend | 640912 |
| B220 | RA3-6B2 | PE | BioLegend | 103207 |
| c-Kit | 2B8 | BV421 | BioLegend | 105828 |
| FoxP3 | FJK-16s | eFluor 450 | Invitrogen | 48-5773-82 |
| IL-4 | 11B11 | PE | BioLegend | 504103 |
| IL-17a | TC11-18H10.1 | APC | BioLegend | 506915 |
| IFN𝛾 | XMG1.2 | FITC | BioLegend | 505805 |
| PD-1 | 29F.1A12 | BV421 | BioLegend | 135217 |
| Propidium Iodide |  | PE-Texas Red (detector) | BioLegend | 421301 |
| Sca-1 | E13-161.7 | PE/Cyanine7 | BioLegend | 122514 |
| SlamF1 | TC15-12F12.2 | PE | BioLegend | 115904 |
| Ter119 | TER-119 | PE/Cyanine5 | BioLegend | 116210 |
| Zombie Red |  | PE-Texas Red (detector) | BioLegend | 423109 |

**Supplemental Figures and Figure Legends**

**SUPPLEMENTARY FIGURE 1**

**Supplementary Figure 1. Gating strategy for Tregs, Tcons, CD8+ T cells, and viability after 18h splenocyte culture in FLU with or without VEN.** **(A)** Representative FACS plots showing gating for percentage of viable Tregs out of total viable CD4+ T cells. Viable CD4+ T cells are first identified by CD3+, CD19-, AmCyan- (to remove autofluorescent cells). Then further separating by CD4+ CD8- cells and gating on PI- and Annexin V- cells to identify viable CD4+ cells. Viable CD4+ T cells are then separated into Tregs and Tcons by comparing FoxP3 and CD25. **(B)** Representative FACS plots showing analyses of Treg, Tcon and CD8+ T cell viability. Total T cells are first identified (CD3+ CD19-), then separated into CD4+ and CD8+ T cells, then within the CD4+ T cells, further separated into total Tregs (FoxP3+) and Total Tcons (FoxP3-). Lastly, PI and Annexin V are compared to identify the percent viability (PI- Annexin V-) in each T cell subtype. **(C)** Representative FACS plots showing Treg viability (PI- Annexin V-) after 18h culture in each condition. **(D)** Representative FACS plots showing CD8+ T cell viability (PI- Annexin V-) after 18h culture in each condition.

**SUPPLEMENTARY FIGURE 2**

**Supplementary Figure 2. Representative FACS plots of all six conditions at all three timepoints.** Representative FACS plots showing **(A)** Percentage of viable (PI- Annexin V-) Tregs (FoxP3+) and Tcons (FoxP3-) out of viable CD4+ T cells. **(B-D)** Percent viability (PI- Annexin V-) of Tcons **(B)**, Tregs **(C)**, and CD8+ T cells **(D)**. The six culture conditions are 1) Vehicle, 2) 3 nM FLU, 3) 10 nM VEN, 4) 100 nM VEN, 5) 3 nM FLU + 10 nM VEN, and 6) 3 nM FLU + 100 nM VEN for 18h.

**SUPPLEMENTARY FIGURE 3**

**Supplementary Figure 3. Representative FACS analysis of cultures without and with IL-2. (A)** FACS of viable (PI- Annexin V-) Tcons (top row), Tregs (middle row), and CD8+ T cells (bottom row) in Vehicle, 3nM FLU, 3nM FLU + 100nM VEN without or with IL-2. **(B)** Representative FACS and gating strategy to measure the viability (PI- Annexin V-) of BM HSCs (Ter119- CD27+ ckit+ Sca1+ SlamF1+ CD34-). The vehicle condition is shown here.

**SUPPLEMENTARY FIGURE 4**

**Supplementary Figure 4. *In vitro* analysis of T cell activation in CD3/CD28 assay or mixed lymphocyte reaction after 6h FV2 culture. (A)** Comparison of percent PD-1+ CD25+ Tcons and CD8+ T cells after 6h culture in Vehicle, FLU, or FLU+VEN+IL-2, followed by 5 day activation with CD3/CD28 antibodies. PD-1 and CD25 expression in unactivated vehicle-treated T cells are also shown. **(B)** Representative FACS and gating strategy to identify activated (PD-1+ CD25+) T cells after 6h conditioning culture followed by 3 days of activation with anti-CD3/CD28 antibodies. **(C-D)** Bar graphs of activated and proliferating (CD25+, CellTrace-) Tcons or CD8+ T cells after 6h FV2 culture followed by 3 days of mixed lymphocyte reaction. Conditions are represented as Vehicle (gray) and FV2 (purple) and as a ratio of responder cells (B6) to stimulator cells (BALB/c). All ratios were calculated according to a scale with a baseline of 1.5 x 10^5^ cells. For example, the 5:5 ratio contained 7.5 x 10^5^ responder cells and 7.5 x 10^5^ stimulator cells. **(E)** Gating schematic for identifying activated proliferating cells following 3 days of MLR. **(F)** Representative FACS plots for activated and proliferating cells in vehicle and FV2 condition after 3 day MLR. Statistics: **(A)** N=2 for activated conditions, 2way ANOVA. * p < 0.033, ** p < 0.002, *** p < 0.001, ns (not significant). **(C-D)** N=5, 2way ANOVA with Bonferroni’s multiple comparisons * p < 0.033, ** p < 0.002, *** p < 0.001, ns (not significant).

**SUPPLEMENTARY FIGURE 5**

**Supplementary Figure 5. Pre-transplant analysis of Treg percentage and post-transplant donor chimerism analysis from haploidentical transplants. (A)** Pre-transplant analysis of donor splenocytes conditioned in either vehicle or FV2 (3nM FLU, 10nM VEN, 20 ng/mL IL-2) after 18h culture. FV2 conditioning increases the Treg percentage approximately 7 fold, from 7.7% (vehicle) to 50.7% (FV2). **(B)** Post-transplant chimerism analysis of FV2 recipients (purple) compared to fresh Hy5 splenocytes. Single, live, CD3-, CD19- cells are then gated on Gr1+ Mac1+ cells to identify the granulocyte population. CFP- (donor FoxC) cells are then identified within the Gr1+ Mac1+ gate. Representative granulocyte (Mac1+ Gr1+, top row) and CFP- (bottom row) FACS plots and gates for Hy5 spleens (left plots) and FV2 recipients (right plots) are shown. Donor chimerism was not assessed in the vehicle recipients as none survived to Week 12. This data demonstrates that the FV2 recipients were successfully engrafted by FV2-conditioned donor cells, and thus the reduction in GVHD was not due to poor engraftment. Statistics: **(A)** Paired T-test (N=3) **(B)** Welch’s T-test (Hy5 spleen N=3, FV2 recipient spleen N=6). * p < 0.033, *** p < 0.001.

**SUPPLEMENTARY FIGURE 6**

**Supplementary Figure 6. Representative FACS plots and gating strategy for analysis of effector T cell subsets 10 days after transplantation with Vehicle- or FV2- conditioned allogeneic grafts. (A)** FACS to identify IFN𝛾+, IL-4+, and IL-17+ Tcons or CD8+ T cells. Spleens from recipients were harvested and stimulated for 4h prior to intracellular staining. FACS plots shown are from a recipient given a vehicle-treated graft. **(B)** FACS to identify donor (H-2^b^+ H-2^d^-) Tregs (CD3+ CD4+ FoxP3+). FACS also identifies donor CD3+ T cells out of total donor cells. Example is from the unstimulated spleen of a recipient given a vehicle-treated graft.
